# Long-term inequalities in health among older Mexican adults: An outcome-wide analysis

**DOI:** 10.1016/j.ssmph.2024.101684

**Published:** 2024-05-24

**Authors:** Aarón Salinas-Rodríguez, Maylen Liseth Rojas-Botero, Ana Rivera-Almaraz, Julián Alfredo Fernández-Niño, Julio César Montañez-Hernández, Betty Manrique-Espinoza

**Affiliations:** a Center for Evaluation and Surveys Research, National Institute of Public Health, Cuernavaca, Morelos, Mexico; b Facultad Nacional de Salud Pública, Universidad de Antioquia, Medellín, Colombia; c Department of International Health, Johns Hopkins Bloomberg School of Public Health, Baltimore, MD, USA

**Keywords:** Longitudinal health inequalities in health, Wealth, Gender, Rural, Geriatric syndromes, Older adults

## Abstract

The relationship between socioeconomic level and health outcomes in older people has been widely studied, but less information about health inequalities associated with gender and place of residence exists. Also, there is scarce evidence of longitudinal inequalities, particularly in countries from the global south. This study aimed to describe the longitudinal patterns of health inequalities associated with wealth, gender, and residence area among older Mexican adults. We used data from two longitudinal studies in Mexico: The Study on Global AGEing and Adult Health (SAGE) and the Mexican Health and Aging Study (MHAS). Three domains to characterize health inequities were used: wealth, gender, and rurality. We conducted an outcome-wide analysis with nine health indicators assessing older adults' physical and cognitive function. The Slope Index of Inequality and the Relative Index of Inequality were used as inequality measurements. Our results indicate that the greatest inequalities are observed in relation to wealth and gender. Older adults with lower socioeconomic status demonstrated higher rates of depression, sarcopenia, falls, and limitations in both basic and instrumental activities of daily living compared to their wealthier counterparts, with increasing trends in physical functionality over time. Furthermore, women experienced higher rates of depression, sarcopenia, frailty, and physical limitations compared to men. The only significant difference related to rurality was a lower rate of frailty among rural older adults. Longitudinal trajectories revealed an increase in the gap of inequality for various health indicators, especially in terms of wealth and gender. Health inequalities in old age are one of the greatest challenges facing health systems globally. Actions like universal coverage of health services for older people and the empowerment of individuals and their communities to have control over their lives and circumstances must be guaranteed.

## Highlights

• A gap persists in understanding longitudinal health inequalities in older populations from the global south.
• We found dynamic inequality patterns in Mexico, with significant changes depression, IADL, sarcopenia, falls, and mortality.
• We uncovered evidence of systematic health inequalities in older Mexican adults, with worse outcomes for the poorest, women, and rural residents.
• Our research underscores the urgent need for universal health services and community empowerment for older adults.

## 1 Introduction

The social and economic context in which people live, from the first years to old age, significantly impacts their health (Katikireddi et al., 2020; Schrempft et al., 2023). The disadvantages experienced since childhood can accumulate and increase the decline in physical and mental abilities in aging. A low socioeconomic level perpetuates these conditions, increasing the risk of death, disability, and even further deterioration in cognitive function (Demakakos et al., 2016; Kok et al., 2016; Makaroun et al., 2017; Steptoe & Zaninotto, 2020). For older people, these economic disadvantages can heighten even further due to decreased income related to retirement or withdrawal from the labor market (Demakakos et al., 2016; Kok et al., 2016), which exacerbates social deprivation and deepens health inequalities (Acciai, 2018). The relationship between socioeconomic level and health outcomes in older people has been extensively studied, reporting higher levels in the prevalence and incidence of physical and mental health conditions in those with lower socioeconomic status (Bodryzlova et al., 2022; Machón et al., 2020; Tsimbos, 2010).

Research has recently been carried out on trends associated with health inequalities, although most have been conducted in high-income countries in the northern hemisphere (Katikireddi et al., 2020; Saravanakumar et al., 2022; Tsimbos, 2010). It has been consistently observed that wealth is strongly associated with mortality throughout adult life (Katikireddi et al., 2020; Makaroun et al., 2017), although some studies suggest that this association tends to attenuate in old age (Huisman, 2004; Reques et al., 2015). Despite this evidence, there has been limited research on the implications of health inequalities for crucial indicators of older people's health, such as geriatric syndromes, which, due to their complexity and interrelationships, substantially impact how we age. For instance, a recent study examined the magnitude of oral health inequalities among older people using longitudinal data from Japan and Singapore, reporting that oral health inequalities increased over time in both countries, but mainly in Singapore (Kiuchi et al., 2023). Nevertheless, studies on health inequalities with older adults have been primarily cross-sectional, and the results have shown that frailty, depression, functional limitations, chronic conditions, falls, low muscle mass, and cognitive impairment are concentrated in older people with a disadvantaged socioeconomic level (Azizabadi et al., 2022; Katikireddi et al., 2020; Makaroun et al., 2017; Salinas-Rodríguez et al., 2019; Saravanakumar et al., 2022; Shang & Wei, 2023; Sharma & Pradhan, 2023; Tsimbos, 2010).

There is less information about health inequalities associated with conditions such as gender and place of residence, which could generate more significant heterogeneity in these inequalities. Even so, women show significant lags concerning men for most of the essential indicators for the health of older people, such as cognition, depression, difficulties performing instrumental activities of daily living, and mobility (Deng & Liu, 2021; Miyawaki & Liu, 2019; Sharma & Pradhan, 2023; Tsimbos, 2010). However, the evidence has been generated in studies that do not necessarily analyze health inequalities. Meanwhile, for men, the risk of premature mortality is higher (Wu et al., 2021). Regarding health inequalities between urban and rural areas, a lower prevalence of cognitive impairment has been reported in urban areas (Sharma & Pradhan, 2023), although other studies did not find differences (Deng & Liu, 2021). A study in China, where 60% of the inhabitants are residents of rural areas, has reported that place of residence could explain health inequalities in favor of residents of urban areas (Jiang et al., 2021). Notwithstanding the above, the studies that have explored potential health inequalities by gender or area of residence have been cross-sectional, and little is known about their trends, except for a few studies conducted in high-income countries (Golinelli et al., 2023; Hammami et al., 2022; Thomson et al., 2018). Even so, less is known of these trends for the older adult population.

Although the educational level and social class have been widely used to analyze health inequalities (Azizabadi et al., 2022; Reques et al., 2015), for older people, income and wealth are measures relative to the individual's ability to purchase resources that, directly or indirectly, produce health benefits and accumulate throughout life (Acciai, 2018; Katikireddi et al., 2020; Tsimbos, 2010). Longitudinal studies analyzing health inequalities focused on wealth among the older adult population have also been scarce. A longitudinal study in 13 European countries of different welfare regimes reported that older people experienced socioeconomic inequalities in quality of life in favor of the more generous welfare regimes (Niedzwiedz et al., 2014). Another longitudinal study in Sweden reported significant inequalities in mortality based on wealth throughout adult life in both men and women (Katikireddi et al., 2020).

Given that significant health inequalities have been identified in countries with higher welfare regimes, it is possible to assume that these inequalities may be even more significant in low- and middle-income countries such as Mexico. Furthermore, Mexico is rapidly aging, and it is estimated that by the year 2050, 24.7% of its population will be 60 years old or older, with a significant proportion of people aged 80 years and older (United Nations, 2015, p. 66). Additionally, a gender gap has been observed for the elderly population in Mexico in the last 20 years regarding life expectancy, healthy life expectancy, and years lived with disability after age 60. Life expectancy has increased in this period for men and women (men: 1990 -21 years; 2019 -23 years; women: 1990 -23 years; 2019 -25 years), although with a lag against men. This same trend is observed for healthy life expectancy (men: 1990 -16 years; 2019 -17 years; women: 1990 -18 years; 2019 -20 years). Meanwhile, for years lived with disabilities, the lag is against women (men: 1990 -18 years; 2019 -19 years; women: 1990 -21 years; 2019 -22 years) (Martinez et al., 2021). Since longitudinal studies on health inequalities with aged populations are limited in low- and middle-income countries, it is essential to generate evidence on trends and trajectories of these inequalities, especially with relevant indicators for this population group, so focused health interventions can be designed for the most disadvantaged groups. In this way, decision-makers can direct resources and efforts in public policy toward the sectors that need it most.

This study aimed to describe the longitudinal patterns of health inequalities associated with wealth, gender, and residence area among older Mexican adults. To this end, data from the two largest cohort studies for older people in Mexico were analyzed: The Mexican Health and Aging Study (MHAS) and the Study on global AGEing and adult health (SAGE-Mexico), as well as relevant health indicators for this population group.

## 2 Material and methods

### 2.1 Data

We used data from the World Health Organization (WHO) Study on global AGEing and adult health (SAGE) in Mexico and the Mexican Health and Aging Study (MHAS). Both studies are described below.

#### 2.1.1 SAGE-Mexico

SAGE is a multi-country, longitudinal study based on nationally representative samples of individuals aged 50+ years in six countries: China, Ghana, India, Mexico, Russia, and South Africa. Details of the study design have been published elsewhere (He et al., 2010). The SAGE-Mexico study and sample (cross-sectional and longitudinal) have been previously described (Miu et al., 2016; Salinas-Rodríguez et al., 2022). Briefly, Wave 1 (baseline data) was collected in 2009 with 2306 respondents. Wave 2 was carried out in 2014 with 2033 interviews (plus 618 new enrolling individuals), Wave 3 in 2017 with 1791 participants (plus 255 new interviews), and Wave 4 in 2021 with 1607 older adults (plus 354 new individuals). The analytical sample consisted of 1484 older adults who had measurements for all waves (Fig. S1, supplementary material). Baseline differences between the final sample and excluded participants were observed. Older adults without follow-up measurements were older, with a lower prevalence of sarcopenia and smoking, mostly women and mainly from rural areas (p < 0.05).

#### 2.1.2 MHAS

MHAS is a longitudinal study with representativeness nationally and by urban-rural strata of older adults in Mexico aged 50 years and over. The baseline survey was conducted in 2001 (n = 15,186) with five follow-up waves: 2003 (n = 14,250), 2012 (n = 18,465), 2015 (n = 15,988), 2018 (n = 18,249), and 2021 (n = 17,538). Details of the study design and objectives have been previously published (Wong et al., 2017). In summary, the study includes direct interviews (selected older adult or her partner, who may be under 50), a proxy, or a next-of-kin. Only direct interviews of individuals aged 50 years or older were considered for this study. The analytical sample consisted of 4288 older adults with measurements for all six waves (Fig. S2, supplementary material). Significant differences were observed between older adults included and those excluded from the study. Those who did not have follow-up measurements were older, with a greater number of chronic conditions, mostly without a partner, and with less health insurance coverage (p < 0.01).

#### 2.1.3 Sample for all-cause mortality in MHAS

For the analysis of all-cause mortality, the entire sample was considered regardless of the number of measurements the older adult had. Given the MHAS design considers incorporating new interviews in each wave, new admissions are left-truncated observations (delayed entry), so not all individuals begin to be at risk simultaneously. Therefore, delayed entry was considered using age as a time scale. Survival time was then defined as the time from age 50 until death or censoring. Table S12 (supplementary material) shows the different settings and their sample size.

### 2.2 Outcomes

#### 2.2.1 Mild cognitive impairment (MCI)

We used an algorithm based on recommendations from the National Institute of Aging and the Alzheimer's Association (Albert et al., 2011), which has been accounted of in a recent study with the SAGE data, to generate the MCI variable (Jacob et al., 2021). The older adults who met all the following criteria were considered to have MCI:

a) Concern regarding a change in cognition. Participants were asked the following questions: “How would you best describe your memory at present?” and/or “Compared to 12 months ago, would you say your memory is now better, the same or worse than it was then?” to evaluate this item. Those OA who reported “bad” or “very bad” and “worse” were considered to have concern with cognition.
b) Evidence of impairment in one or more cognitive domains were assessed (based on a <−1 SD cut-off after adjustment for level of education and age) with immediate and delayed verbal recall, forward and backward digit span and verbal fluency tests.
c) Independence in basic activities of the daily life (BADL) evaluated with Katz scale (Katz et al., 1963).
d) Not demented participants who could not take the survey due to a severe cognitive impairment. A close family member or caregiver reported whether the older adult had frequent episodes of memory loss or spatial/temporal disorientation.

#### 2.2.2 Depression

We assessed depression through a set of symptomatic questions based on the World Mental Health Survey version of the Composite International Diagnostic Interview (Kessler & Ustün, 2004). Diagnosis of a major depressive episode was assessed based on the severity of reported symptoms of depression during the past 12 months (Ayuso-Mateos et al., 2010). The detailed symptomatic questions and algorithm have been provided in previous research using the SAGE dataset (Arokiasamy et al., 2015).

#### 2.2.3 Sarcopenia

The presence of sarcopenia was defined, according to previous studies using the Mexico-SAGE data (Salinas-Rodríguez et al., 2021), as having low skeletal muscle mass (SMM), reflected by lower skeletal muscle mass index (SMI), and either or both slow gait speed and weak handgrip strength. Specifically, sarcopenia was determined according to the following criteria:

a) Low skeletal muscle mass. First, SMM was calculated as the appendicular skeletal muscle mass (ASM) based on the equation proposed by Lee et al. (Lee et al., 2000). Further, the SMI was obtained by dividing the ASM by the body mass index (BMI) (Studenski et al., 2014). Then, low SMM (defined as the presence of low SMI) was established by the lowest quintile of the SMI based on sex-stratified values.
b) Slow gait speed. It was defined as the lowest quintile of walking speed (m/s) based on height, age, and sex-stratified values (Capistrant et al., 2014).
c) Weak handgrip strength. Using the average value of the two handgrip measurements of the dominant hand, weak handgrip was defined as <27 kg for men and <16 kg for women (Cruz-et al., 2019).

#### 2.2.4 Frailty

Frailty status was determined using the frailty phenotype, based on the criteria proposed by Fried et al. (Fried et al., 2001), which covers five components: weight loss, exhaustion, low physical activity, slow walking speed and weakness. Respondents were considered frail if they met three or more of these criteria, prefrail if they met one or two, and not frail or robust if they met none of the above criteria. Since the original cut-off points of the frailty phenotype have not been validated in low- and middle-income countries, we used the lowest quintile approach (country-specific) for the items measured on a continuous scale (Saum et al., 2012). Details of the application on this measurement of frailty in the SAGE sample have been published elsewhere (Hoogendijk et al., 2018). Briefly described, gait speed was measured by recording the time taken in seconds to walk 4m at a normal speed. Slow gait speed was defined by the lowest quintile, stratified by sex and height. The presence of the weight loss criterion was based on the lowest quintile of body mass index (BMI). Grip strength was assessed with a handheld dynamometer, using the sum of the highest values of two measurements on each hand. The lowest quintile stratified by sex and BMI was applied as a cut-off to indicate low grip strength. Exhaustion was measured on a 5-point Likert scale by asking respondents whether they had enough energy for daily activities. This criterion was considered present if participants answered, “Not at all” or “A little”. Finally, physical activity was assessed using the WHO Global Physical Activity Questionnaire (GPAQ). The low physical activity criterion was present if activity<600 MET-minutes a week as defined by the GPAQ.

#### 2.2.5 Quality of life (QoL)

We assessed this variable using the WHOQOL (WHO Quality of Life) instrument. This eight-item questionnaire covers the following core domains (two items per domain): physical, psychological, social, and environmental. The eight items are summed for an overall score ranging from 0 to 100. The higher the score, the higher QoL (Skevington et al., 2004).

#### 2.2.6 Basic activities of daily living (BADL)

The activities proposed by the Katz index were considered to evaluate the ability to perform the following basic functions without difficulty or help: bathing, dressing, using the toilet, moving around the home, feeding, and incontinence (Katz et al., 1963).

#### 2.2.7 Instrumental activities of daily living (IADL)

The activities that comprise the Lawton and Brody index were analyzed to evaluate the ability to perform the following functions without difficulty or help: preparing food, taking medications, shopping, and managing money (Lawton & Brody, 1969).

The difficulty and need for help from another person to perform at least one of the activities analyzed in each scale was considered a limitation in BADL and IADL.

#### 2.2.8 Falls

The question “Have you fallen in the last two years?” was used to determine if the older adult experienced one or more falls in the two years prior to the survey.

#### 2.2.9 All-cause mortality

Data on death (from any cause) were obtained from a next-of-kin or proxy interview along with the date of death (month and year).

### 2.3 Exposures

We used three domains to characterize health inequities: socioeconomic position (SEP), gender, and rurality.

#### 2.3.1 SEP

A household wealth index was derived using the WHO standard approach to estimate permanent income from the ownership of durable goods, dwelling characteristics (type of floors, walls, and cooking stove), and access to water, sanitation, and electricity services (Howe et al., 2012). The index was transformed into quintiles, with the lowest quintile (Q1, reference category) indicating the poorest households and the highest quintile (Q5) the richest.

#### 2.3.2 Gender

A dichotomous variable was formed, with the male being the reference category.

#### 2.3.3 Rurality

According to the area of residence of the older adults, a dichotomous variable was defined: rural for ≤ 2500 people and urban for >2500 people. The urban area was the reference category.

### 2.4 Covariates

The following health and socioeconomic variables were included: age, union status (with partner = 1), having a paid job, and health insurance (yes = 1). The number of chronic conditions was defined as follows. For SAGE-Mexico, we used the list of eight chronic diseases. The following conditions were measured according to self-reported medical diagnoses: diabetes, stroke, and cataracts. Another four conditions were estimated through algorithms for symptomatology and self-reported treatment: angina, arthritis, chronic obstructive pulmonary disease, and asthma. Either blood pressure measurement or self-reported treatment determined hypertension. A detailed description of the definition and operationalization of these diseases has been published elsewhere (Arokiasamy et al., 2015). The self-reported medical diagnosis of diabetes, hypertension, cancer (any type), asthma, heart attacks or heart failure, stroke, and arthritis were included in MHAS. In both cases, the number of chronic conditions was expressed as a count variable that resulted from the arithmetic addition of all the conditions considered.

### 2.5 Statistical analysis

The baseline characteristics of the participants were described using means, standard deviations (SD), and proportions where appropriate. We used Chi-square or t-student tests to compare the health and sociodemographic characteristics according to the exposure variables.

We used two indices to estimate the magnitude of socioeconomic inequalities related to exposure variables. The Slope Index of Inequality (SII) measures absolute inequality, reflecting health outcome variations between the reference category and other stratifying categories related to equity. When SII is positive, comparison groups exhibit worse health outcomes than the reference category; conversely, a negative SII suggests that comparison groups have better health outcomes than the reference category. Conversely, the Relative Index of Inequality (RII) is a relative measure of inequality. An RII distinct to 1 signals the existence of health inequalities. Specifically, an RII less than 1 suggests that comparison groups exhibit better health outcomes than the reference category, whereas an RII greater than 1 implies worse outcomes than the reference category. Zero value for SII and 1 for RII indicates nonsignificant health outcome inequalities between comparison groups (Kakwani et al., 1997; Khang et al., 2008).

Since we had repeated measurements, we applied mixed-effects generalized linear models (ME-GLM) with an identity link function to calculate SII, and a logarithmic link function to calculate RII. Specifically, we used the binomial family for dichotomous data, gaussian for continuous data, and Poisson for mortality data. In the first case, the estimated parameters are interpreted as rate differences and rate ratios in the second one (Kakwani et al., 1997; Khang et al., 2008). For each indicator, the SII and RII were estimated as follows: wealth, quintile 5 (richest) vs. quintile 1 (poorest); gender, female vs. male; and rurality, rural vs. urban. We also depicted graphically the longitudinal inequalities related to the outcomes for each exposure variable, using the conditional expected value (probability or mean) obtained from the ME-GLM. Additionally, for the analysis of all-cause mortality, we incorporated the delayed entry feature in our statistical analysis following Lamarca et al.'s (Lamarca et al., 1998) proposal to analyze left-truncated data with the older adult population using age as the time scale.

Since we sought to identify possible variations in trends in health inequalities, the regression models included a multiplicative interaction term between follow-up time and exposure variables (wealth, gender, and rurality). Likewise, the wave-specific centered age was included as a covariate to minimize collinearity issues and untangle the effects of time and age. All models were adjusted using Stata v18.0 (StataCorp. 2023. Stata Statistical Software: Release 18. College Station, TX: StataCorp LLC.).

## 3 Results

The analytical sample of SAGE-Mexico was constituted of 1484 older adults. At baseline, 60.6% were female, and the mean age was 65.5 (SD = 14.2). 13.4% had MCI, 8.4% had depression, 15.7% had sarcopenia, and 9.9% were frailty (Table 1). Women had a higher prevalence of depression (p < 0.01), frailty (p < 0.01), and lower quality of life (p < 0.01). There were no significant differences in MCI and sarcopenia (Table 1). The data with the distribution of outcomes and covariates by wealth and rurality are shown in Tables S1 and S2 of the supplementary material. Regarding MHAS, 4288 older adults were included. At baseline, 57.4% were female, and the mean age was 57.6 (SD = 6.1). 4.4% had limitations in BADL and 3.7% in IADL, and 33.7% had at least one fall in the last 12 months. Women had a higher prevalence of limitations in BADL (p < 0.01), IADL (p < 0.01), and falls (p < 0.01) (Table 2). Tables S3 and S4 (supplementary material) show the data by wealth and rurality.

**Table 1:** Baseline health and sociodemographic characteristics by sex, SAGE-Mexico.

Variable	Total n = 1484	Male n = 585	Female n = 899	p-value^a^
**Outcomes**				
Mild cognitive impairment	13.4	12.0	14.4	0.14
Depression	8.4	4.7	10.8	<0.01
Sarcopenia	15.7	15.6	15.8	0.96
Frailty	9.9	7.3	11.6	<0.01
Quality of life	65.5 (14.2)	66.9 (13.6)	64.5 (14.4)	<0.01
**Covariates**				
Age	68.4 (9.5)	68.4 (9.5)	68.4 (9.5)	0.97
Number of chronic conditions	2.1 (1.6)	1.8 (1.5)	2.3 (1.7)	<0.01
Paid job	25.1	42.0	14.0	<0.01
Union status (with partner)	61.0	79.7	48.8	<0.01
Health insurance coverage	70.0	68.7	71.0	0.29

Values in cells are means (std. dev.) or percentages.

a 2 proportions or t-student tests.

**Table 2:** Baseline health and sociodemographic characteristics by sex, MHAS.

Variable	Total	Male	Female	p-value^a^
	n = 4288	n = 1825	n = 2463	
**Outcomes**				
BADL limitations	4.4	2.9	5.5	<0.01
IADL limitations	3.7	1.9	5.0	<0.01
Falls (at least one in last two years)	33.7	24.5	40.5	<0.01
**Covariates**				
Age	57.6 (6.1)	57.6 (6.1)	57.7 (6.1)	0.49
Number of chronic conditions	0.7 (0.8)	0.5 (0.7)	0.9 (0.9)	<0.01
Paid job	52.3	83.0	29.7	<0.01
Union status (with partner)	75.9	89.3	66.4	<0.01
Health insurance coverage	59.0	55.3	61.7	<0.01

BADL: basic activities of daily living; IADL: instrumental activities of daily living.

Values in cells are means (std. dev.) or percentages.

a 2 proportions or t-student tests.

Table 3 shows the descriptive statistics for the analysis of all-cause mortality. The median follow-up time was 35 years, and the mortality rate was 14.7 per 1000 person-years. The poorest older adults had a higher mortality rate compared to the richest (p < 0.01), as well as men compared to women (p < 0.01). There were no differences in the rural vs. urban comparison.

**Table 3:** Descriptive survival and mortality data by sex, wealth, and rurality, MHAS.

	Time at risk (person-years)	Mortality rate (per 1000)	Participants	Survival time (years)			p-value^a^
				25%	50%	75%	
**Wealth**							
Q1	77,628	15.8	3680	28	35	42	
Q5	166,011	13.4	7693	28	36	43	<0.01
**Sex**							
Men	232,234	15.9	11,376	26	34	41	
Women	253,810	13.6	12,377	28	36	43	<0.01
**Dwelling area**							
Urban	394,796	14.7	19,453	27	35	42	
Rural	91,266	14.8	4300	28	35	42	0.85
Total	486,062	14.7	23,753	27	35	42	

a Incidence-rate ratio test.

Tables S5-S7 (supplementary material) depict the estimated inequalities in health for the three domains analyzed: wealth, gender, and rurality for SAGE-Mexico. Significant wealth inequalities were observed for depression, sarcopenia, and QoL. Roughly, individuals with the highest wealth (Q5) had lower rates of depression, sarcopenia, and higher QoL than poorer older adults (Q1) over time. There were also significant longitudinal inequalities by gender. Women had higher rates of depression, sarcopenia, and frailty. For rurality, the only significant difference was observed for frailty, with rural older adults having lower rates than urban counterparts.

For the MHAS, Tables S8-S11 (supplementary material) display the results for the inequalities in health. The data indicate that wealthier individuals had lower rates of falls, BADL and IADL over time than poorer older adults. Concerning gender, women had higher rates than men in the same three outcomes. No significant differences were observed according to rurality. Regarding all-cause mortality, men and those living in urban areas had higher rates than women and those living in rural areas. There were no significant differences according to wealth.

**Fig. 1:**
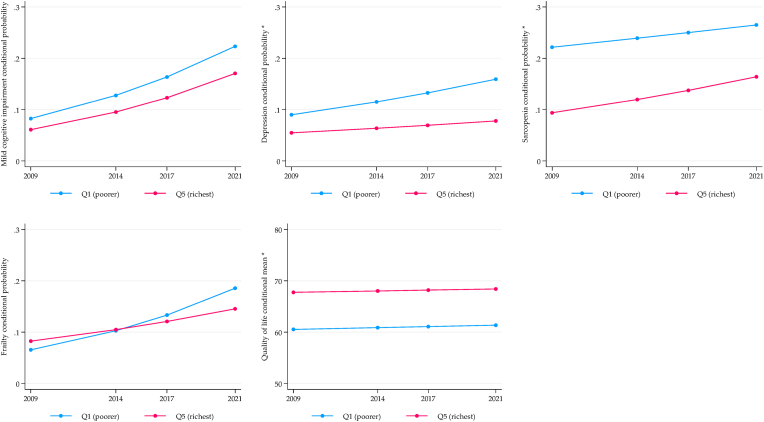
Longitudinal wealth inequalities, SAGE-Mexico.

**Fig. 2:**
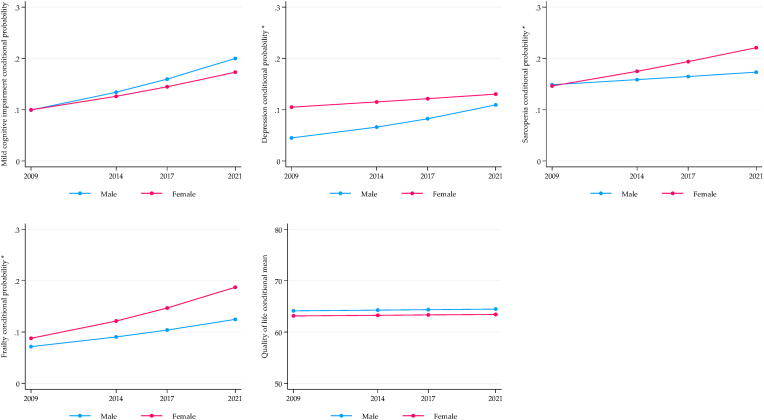
Longitudinal sex inequalities, SAGE-Mexico.

**Fig. 3:**
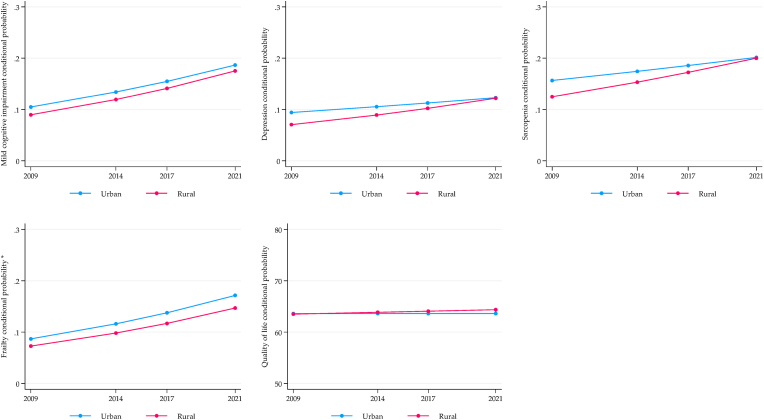
Longitudinal rural/urban inequalities, SAGE-Mexico.

Fig. 1, Fig. 2, Fig. 3 display the longitudinal trajectories of health inequalities for SAGE-Mexico using the conditional expected value (probability or mean) obtained from the ME-GLM. For the wealth indicator, mixed results were observed. For depression, there is a significant gap between the richest and the poorest, with an increasing trend after 12 years of follow-up. Frailty showed no significant differences in the baseline, but a gap in favor of the richest was observed in the fourth wave (2021). On the contrary, the gap was reduced for sarcopenia, but the inequality persists. The quality-of-life gap did not change, and inequality against the poorest persisted (Fig. 1).

Related to gender, sarcopenia and frailty showed an increase in the inequality gap disadvantaging women, although no differences were observed at baseline. The trend for depression was decreasing, with no differences in the fourth wave (2021). No significant inequalities were observed for MCI and quality of life. However, there was a significant increase in the rate of MCI in men and women while quality of life remained stable (Fig. 2). The only significant gap in the rural vs. urban comparison was the related to frailty, which remained constant over time and to the detriment of urban older adults (Fig. 3).

**Fig. 4:**
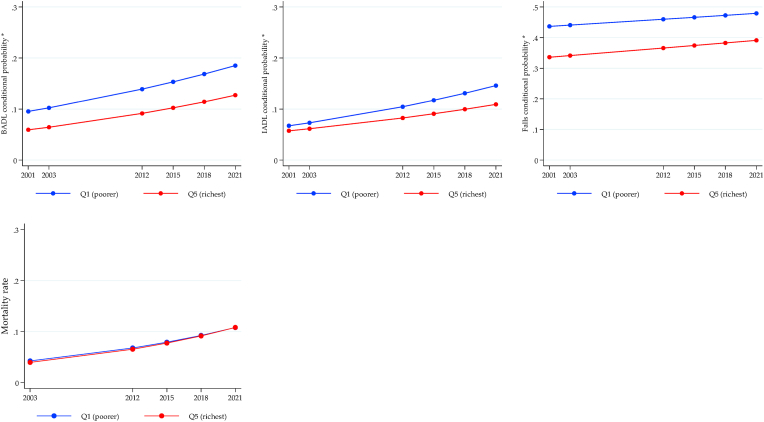
Longitudinal wealth inequalities, MHAS.

**Fig. 5:**
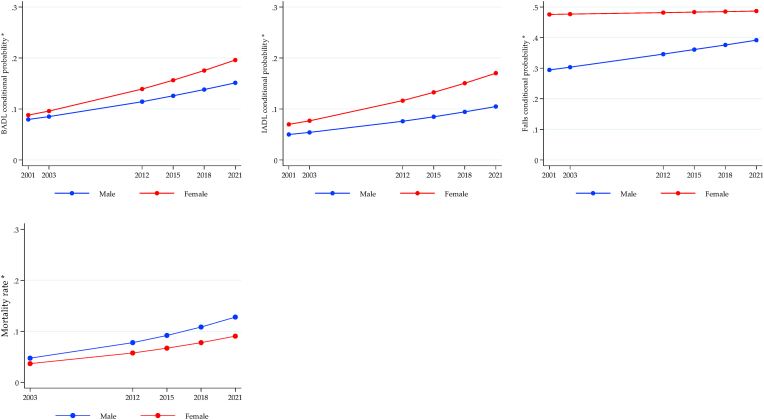
Longitudinal sex inequalities, MHAS.

**Fig. 6:**
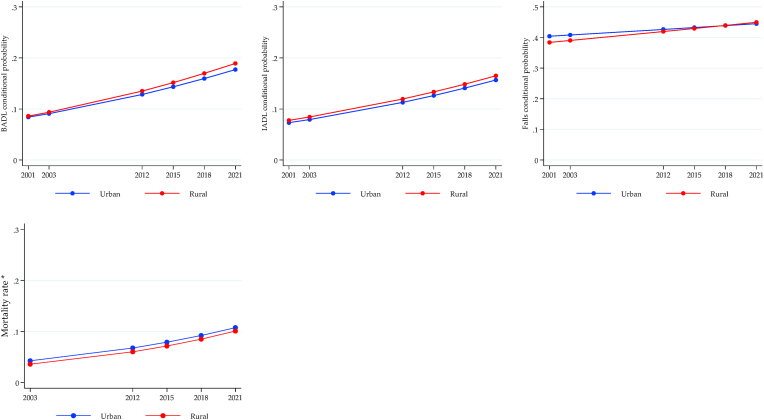
Longitudinal rural/urban inequalities, MHAS.

The longitudinal trajectories of health inequalities for MHAS are shown in Fig. 4, Fig. 5, Fig. 6, again using the conditional expected value from the ME-GLM. Significant gaps were observed for physical functionality outcomes (BADL, IADL, and falls) to the detriment of the poorest older adults, with an increasing trend for BADL and IADL. There were no differences in the mortality rate (Fig. 4). Gender inequalities are shown in Fig. 5. The gap in BADL and IADL increased significantly, negatively impacting women. While the gap in falls decreased, although it remained against women. On the contrary, the gap in the mortality rate increased unfavorably for men. Finally, all-cause mortality was the only outcome where a significant gap was observed in the rural vs. urban comparison, with slightly higher rates in urban areas but constant over time (Fig. 6).

## 4 Discussion

### 4.1 Main findings

The present study analyzed health inequalities associated with wealth, gender, and area of residence in a broad set of health outcomes for older adults using longitudinal and representative data from SAGE-Mexico and MHAS. The results provide robust evidence of significant inequalities related to nine critical health indicators for this population group, including some geriatric syndromes such as frailty, sarcopenia, and mild cognitive impairment. Additionally, the results reveal that, in general, inequalities associated with wealth, gender, and area of residence persist over time and, in some cases, have widened. These results are relevant for Mexico but also for other low- and middle-income countries (even for some high-income ones) since offer novel evidence on the persistence of structural inequities for the older adult population.

The results of this study could be of interest in low- and middle-income countries such as Mexico, where there has been an accelerated process of population aging that coexists with high levels of poverty and limited access to health services and social well-being. Even so, during the last 40 years, Mexico has implemented social policies to alleviate poverty and reduce the gaps of historically disadvantaged groups (poor people, women, indigenous people, etc.). In particular, and for the older adult population, conditional transfer programs (Salinas-Rodríguez et al., 2013) have been implemented, as well as programs to increase the coverage of health services (Rivera-Hernandez & Galarraga, 2015), reduce food insecurity (Barquera et al., 2001), and improve the income of older people through non-contributory pensions (Salinas-Rodríguez et al., 2014). This fact could explain why some of the gaps observed in this study have remained constant and have not increased over time.

### 4.2 Comparison with findings from previous studies

It is difficult to compare our results because no other study had a similar scope. We chose four studies that are quite different but relevant for triangulation, all generated in high-income and northern hemisphere countries. Of these studies, only two reported a specific measure of inequality (RII); the others reported results without an analytical approach to inequalities and incorporated some measure of wealth or income as an independent variable in their analyses.

Our results showed a constant (significant) trend in the differences in inequalities by gender and rurality for all-cause mortality over a 20-year follow-up period. While significant differences were observed by wealth quintiles for the period 2001–2012, but faded for the period 2013–2021. Two previous studies have analyzed inequality in mortality by wealth using data from public government records combined with data from representative surveys and reporting RII as a measure of inequality.

In Sweden, Katikireddi et al. analyzed data from 6 million adults aged 25 and over, of which 1.1 million were over 55 (Katikireddi et al., 2020). Mortality was estimated for 17 years of follow-up (1990–2007), and wealth was measured by the amount of wealth tax paid by an individual at baseline measurement (1990). A measure of individual net disposable income (total income from paid employment and benefits minus taxes) and household income (equivalized for household composition) was also included. The analysis was stratified by sex and age groups (25–39, 40–54, 55–64, 65–74, 75–84 and 85+ years). Their results showed greater inequality (poorest vs. richest) in mortality for both men (RIIs varying from 1.15 to 2.76) and women (RIIs varying from 1.08 to 2.31).

Reques et al. reported inequalities in mortality for almost 7 million older Spanish adults (65+) during a 7-year follow-up (Reques et al., 2015). As indicators of socioeconomic level, they used the educational level and three other indicators that reflect material wealth: number of rooms in the home, surface area of the home in square meters, and number of cars owned by the home's inhabitants. Analyzes were stratified by sex and age groups (65–74, 75–84, and 85+ years). The results also showed greater inequality (poorest vs. richest) in mortality for men (RIIs varying from 1.05 to 1.86) and women (RIIs varying from 1.03 to 1.62).

Despite the marked differences with our study, our results are consistent since poorest individuals had a greater disadvantage than the richest (RIIs varying from 1.07 to 1.15). However, there are significant methodological differences. First, Katikireddi et al. and Reques et al. analyze inequalities in mortality based on baseline wealth data and, therefore, do not show the trend of these inequalities. Second, they focused only on economic inequalities, while we explore inequalities by gender and rurality. Third, we used one measure of wealth (permanent household income); while these studies explore other measures (income from taxes, educational level, and material wealth); despite this, the results were consistent.

Two studies have analyzed the relationship between socioeconomic level and broad definitions of successful aging. However, neither applied an analytical approach to estimate health inequalities and, therefore, did not report inequality measures (SII or RII). Kok et al. reported the results of a study with longitudinal data from 16 years of follow-up with 2095 older adults (between 55 and 85 years at baseline measurement, 1992) from the Longitudinal Aging Study Amsterdam (Kok et al., 2016). They defined nine indicators of successful aging (functional limitations, self-rated health, cognitive functioning, depressive symptoms, satisfaction with life, social loneliness, emotional support given, instrumental support given, and social activity) and generated an index of successful aging (range 0–9). The results showed that a higher educational level, a higher level of occupational skills, and a higher income were associated with successful aging trajectories. Steptoe and Zaninotto analyzed data from 5018 participants (aged 52+ years) from the English Longitudinal Study of Aging (ELSA) assessed in 2004 (Wave 2) and eight years later in 2012 (Wave 6) to determine the relationship between low socioeconomic status and the acceleration of aging (Steptoe & Zaninotto, 2020). They used 19 health outcomes grouped into six domains (physical capability, sensory function, markers of physiological function, cognitive function, emotional well-being, and social function) related to accelerated aging. The authors concluded that lower socioeconomic status is related to accelerated aging across a wide range of functional abilities and phenotypes independently of the presence of health conditions.

The results of our study have shown significant longitudinal socioeconomic inequalities for six key outcomes associated with aging: depression, sarcopenia, functional limitations (BADL and IADL), falls, and quality of life. These results coincide with those of Kok et al. (Kok et al., 2016) and Steptoe & Zaninotto (Steptoe & Zaninotto, 2020) in that poorer older people exhibit worse conditions than their wealthier counterparts. Although, once again, these studies are only partially comparable with ours. First, Kok et al. and Steptoe & Zaninotto do not determine the presence of significant inequalities but rather report significant associations between wealth and various health outcomes. Second, although they analyze longitudinal data, no trends in inequalities are identified. Third, inequalities by gender and rurality are not analyzed.

### 4.3 Explanations of our findings

When analyzing socioeconomic health inequalities in aging, it is essential to consider two contrasting and widely known hypotheses to understand the effects of inequality on health across the lifespan. First, the "cumulative effect hypothesis" posits that health inequalities may widen with age, given the cumulative influence of social, economic, and demographic advantages or disadvantages over the lifespan (Ferraro & Shippee, 2009). Conversely, the "age leveler hypothesis" suggests that health inequalities may decrease because aging leads to increased health risks, multimorbidity, and frailty, regardless of determinants such as sex, socioeconomic level, and place of residence (Rehnberg, 2020).

In this study, we observed a progressive trend of deterioration in all health outcomes evaluated, except quality of life. Older adults showed an increased probability of presenting mild cognitive impairment, depression, sarcopenia, frailty, BADL, IADL, falls, and all-cause mortality. After adjusting for covariates, we found that inequalities based on wealth persisted into old age, consistent with the cumulative effect hypothesis. Regarding socioeconomic position, it has been widely described that wealth plays a determining role in the distribution of health and disease at the population and individual levels throughout the life course (Braveman, 2022). Individuals with different socioeconomic levels experience different health trajectories over time, with those in less privileged socioeconomic conditions experiencing worse health outcomes, higher morbidity, and premature mortality compared to those in better socioeconomic conditions, evidence that has been reported consistently from birth to adulthood (Varghese et al., 2021). However, the influence of socioeconomic level on health is less evident at advanced ages, among other reasons, because there are divergent findings on the relationship between socioeconomic status and health in old age (Acciai, 2018).

Our research highlights that gender plays a pivotal role in shaping health disparities, showing that gender-linked health differences persist and tend to intensify as individuals age (Newman & Brach, 2001). Women consistently exhibit worse conditions such as depression, sarcopenia, frailty, BADL, IADL, and falls than men. These results suggest a more profound degree of disability in women. Earlier research supports our findings, indicating that older women are more predisposed to disability than men (Millán-Calenti et al., 2010). As discussed recently, two primary mechanisms could account for gender-based health disparities (Salinas-Rodríguez et al., 2020). The first pertains to the higher prevalence of some chronic conditions in women, which escalate the risk of complications in old age, such as sarcopenia, frailty, and falls (Salinas-Rodríguez et al., 2014). The second highlights the socioeconomic inequalities associated with gender. International studies, including those from the global south, show that a buildup of social challenges, ranging from limited educational access and lower income levels to traditional caregiving duties, disproportionately affects women (Hoogendijk et al., 2008; Hosseinpoor et al., 2012). The adverse health implications of these socio-economic barriers intensify as women age.

Regarding the comparison by residence area, a sustained increase in urbanization has caused people in urban areas to be exposed to a great diversity of social, physical, and environmental risk factors. At the same time, urban areas offer greater availability and access to services, including health services (Muhammad, 2023). This duality makes the residence area an essential axis to investigate the health inequalities throughout the life cycle, including old age. This issue is particularly relevant for Mexico, where the trend towards urbanization has been increasing since the 1950s, reflected in the fact that, by 2020, 79% of its population lived in urban areas (INEGI. et al., 2023). However, in most of the outcomes, no inequalities related to the area of residence were detected. This fact aligns with the hypothesis of leveling with age or suggests that the initial differences were confounded by characteristics such as socioeconomic level. Even so, urban older adults had a significantly higher risk of frailty and all-cause mortality than rural ones. These results could be explained by less healthy lifestyles in urban areas, like greater physical inactivity, unhealthy diets, higher alcohol consumption, more psychosocial stress, and exposure to environmental pollution. However, it is also possible to have a "survival filter" effect in rural areas, given that only the healthiest individuals reach old age, as opposed to urban areas where individuals with chronic health conditions reach old age due to better access to health services throughout life.

### 4.4 Limitations

Our study has some limitations that should be considered when interpreting the findings. First, it is not possible to make causality conclusions because even after adjustment for covariates, there may still be residual confounding. The determinants analyzed here operate, directly and indirectly, on outcomes through intricate mechanisms that cannot be identified in a single study, given that our objective was to carry out a multidimensional exploration of health inequalities. However, it is worth highlighting that the exposures analyzed tend to vary little over time, like belonging to the extreme levels of wealth distribution, and even not vary like sex. Therefore, it is reasonable to assume that the exposures temporally preceded the outcomes, and therefore, the cumulative effects of these determinants during old age can be estimated. Second, although our data sources are nationally representative, mortality selection and survivor bias may lead to study results that are not necessarily representative and probably not extrapolated to populations with marked differences in premature mortality. Third, some health measures analyzed are based on self-reports, which could introduce a recall bias. However, in many cases, this is due to the very nature of the constructs, and the error is expected to be non-differential due to the strict protocol of both studies. Finally, we used biological sex as a proxy of gender identity. Although our statistical models included sex, the lack of variables that address broader aspects of gender may limit our understanding of more intricate inequalities related to gender roles, identities, and relationships.

### 4.5 Strengths

Likewise, this study has strengths relevant to increasing global evidence on inequalities among older adults. First, it represents a significant advance in adopting a longitudinal approach to assess social inequalities in health at the individual level in low- and middle-income countries. In contrast, longitudinal analyses of inequalities have been conducted adopting an ecological perspective or with territorial units of analysis. Second, we have used two important sources of information, both with repeated measurements, for older adults in a global south country. So, data from 5700 older adults in 12 years follow-up for the SAGE-Mexico and 21 years for MHAS has been analyzed. Third, we have carefully selected indicators relevant to the health of older adults focused on aspects of functionality and quality of life, as opposed to conventional indicators that focus on the prevalence of chronic diseases and premature mortality in targeted health analyses of younger populations. Fourth, the choice of wealth as a stratification measure for analyzing inequality. Evidence has shown that wealth is the most relevant indicator for analyzing health inequalities for the older adult population. Unlike income, which can be volatile and decline after retirement, wealth represents the accumulation of economic advantages over a lifetime. Furthermore, wealth may reflect forward-thinking and cautious behavior, crucial resources in old age (Acciai, 2018).

## 5 Conclusions

The longitudinal analysis presented in this study underscores the persistent and complex health inequalities among older adults, significantly influenced by wealth, gender, and area of residence. These findings carry profound implications for both the scientific community and public health policymakers. From a research perspective, our study highlights the necessity of adopting a multidimensional approach in the examination of health disparities in aging populations. Health inequalities in older adults are not merely the result of individual lifestyle choices but emerge from a complex interplay of socioeconomic factors, gender dynamics, and environmental contexts. This complexity mandates a comprehensive analytical framework that accounts for the structural determinants of health, including economic conditions, social roles, and geographic accessibility to healthcare services.

For public health policy, the evidence points towards the urgent need for tailored interventions that address the root causes of health inequalities. Universal access to healthcare and coverage must be more than just policy aspirations (MacGuire, 2020); they need to be actionable priorities that take into account the varied needs of older adults based on their economic status, gender, living conditions and territory. This approach requires a departure from traditional, individualistic health interventions towards more inclusive strategies that empower older individuals and their communities. Such strategies should foster environments that enable older adults to maintain their health, dignity, and autonomy, in line with the WHO's vision of healthy aging (Menassa et al., 2023).

Our findings provide evidence for policymakers on the importance of intersectoral management of social determinants throughout the life course. The disparities highlighted in this study call for concrete actions, including promoting physical activity among urban dwellers, ensuring a basic living income, reducing income inequality, enhancing opportunities for women, and mitigating the oppression of the patriarchal system. These intersectoral actions must move beyond rhetoric to manifest in plans, projects, and programs with allocated resources, specific targets, and accountability.

Moreover, our findings advocate for targeted policies that specifically address the needs of the most vulnerable subgroups identified in our study—women, the economically disadvantaged, and those residing in urban areas—to mitigate the disparities observed. Ensuring these populations have equitable access to preventive and curative health services is critical.

Advancing health equity among older adults necessitates a concerted effort that integrates robust research with innovative public health policies. By acknowledging and addressing the intricate factors contributing to health inequalities, we can aspire to a future where healthy aging is a feasible reality for all, irrespective of wealth, gender, or residence.

## Data access statement

The raw datasets analyzed during the current study are available in the following repositories:

## SAGE

https://www.who.int/data/data-collection-tools/study-on-global-ageing-and-adult-health/sage-waves.

## MHAS

https://www.mhasweb.org/DataProducts/Home.aspx.

## Ethical statement

All procedures performed involving human participants were in accordance with the ethical standards of the 1964 Helsinki declaration and its later amendments or comparable ethical standards. Written informed consent was obtained from all participants in the study. SAGE-Mexico study protocol was approved by the Ethics Committee of the National Institute of Public Health (INSP), Mexico. The study protocol and instruments of MHAS were approved by the Institutional Review Board or Ethics Committee of the University of Texas Medical Branch, the National Institute of Statistic and Geography (INEGI) and INSP in Mexico.

## Financial disclosure statement

This research did not receive any specific grant from funding agencies in the public, commercial, or not-for-profit sectors.

## Funding

SAGE is supported by 10.13039/100004423WHO and the 10.13039/100000049US National Institute on Aging through Interagency Agreements (OGHA04034785, YA1323-08-CN-0020, and Y1-AG-1005-01) and competitive grant: R01AG034479. The MHAS (Mexican Health and Aging Study) is partly sponsored by the 10.13039/100000002National Institutes of Health/10.13039/100000049National Institute on Aging (grant number NIH R01AG018016) in the United States and the Instituto Nacional de Estadística y Geografia (INEGI) in Mexico.

## CRediT authorship contribution statement

**Aarón Salinas-Rodríguez:** Conceptualization, Formal analysis, Investigation, Methodology, Visualization, Writing – original draft, Writing – review & editing, Supervision, Data curation. **Maylen Liseth Rojas-Botero:** Conceptualization, Investigation, Methodology, Writing – original draft, Writing – review & editing. **Ana Rivera-Almaraz:** Investigation, Writing – original draft, Writing – review & editing. **Julián Alfredo Fernández-Niño:** Methodology, Writing – original draft, Writing – review & editing, Conceptualization, Investigation. **Julio César Montañez-Hernández:** Data curation, Formal analysis, Investigation, Visualization, Writing – original draft, Writing – review & editing. **Betty Manrique-Espinoza:** Conceptualization, Investigation, Methodology, Writing – original draft, Writing – review & editing.

## Declaration of competing interest

The authors declare that they have no known competing financial interests or personal relationships that could have appeared to influence the work reported in this paper.

## Data Availability

The raw datasets analyzed during the current study are available in the following public repositories. Links are provided.
