# Supplementary material for: Long-term inequalities in health among older Mexican adults: An outcome-wide analysis
Source: SSM Popul Health. 2024 May 24;26:101684. doi: 10.1016/j.ssmph.2024.101684 (PMC11179325; doi:10.1016/j.ssmph.2024.101684)
Supplement: Multimedia component 1 [file mmc1.docx]

**Table S.1. Baseline health and sociodemographic characteristics by wealth, SAGE-Mexico**

| **Variable** | **Total**  **N=1484** | **Q1**  **(poorer)**  **n=494** | **Q5**  **(richest)**  **n=346** | **p-value*** |
| --- | --- | --- | --- | --- |
| **Outcomes** |  |  |  |  |
| Mild cognitive impairment | 13.4 | 12.2 | 8.3 | 0.08 |
| Depression | 8.4 | 7.9 | 6.1 | 0.26 |
| Sarcopenia | 15.7 | 21.5 | 10.6 | <0.01 |
| Frailty | 9.9 | 11.8 | 7.9 | 0.04 |
| Quality of life | 65.5 (14.2) | 61.6 (14.0) | 70.0 (13.8) | <0.01 |
| **Covariates** |  |  |  |  |
| Age | 68.4 (9.5) | 69.1 (9.6) | 66.2 (9.5) | <0.01 |
| Number of chronic conditions | 2.1 (1.6) | 1.8 (1.4) | 2.2 (1.6) | <0.01 |
| Paid job | 25.1 | 26.9 | 28.7 | 0.64 |
| Union status (with partner) | 61.0 | 53.6 | 62.2 | 0.01 |
| Health insurance coverage | 70.0 | 59.1 | 77.7 | <0.01 |

* 2 proportions or t-student tests.

Values in cells are means (std. dev.) or percentages.

Total is the analytical sample size, not Q1+Q5.

**Table S.2. Baseline health and sociodemographic characteristics by dwelling area, SAGE-Mexico**

| **Variable** | **Total**  **N=1484** | **Rural**  **n=585** | **Urban**  **n=899** | **p-value*** |
| --- | --- | --- | --- | --- |
| **Outcomes** |  |  |  |  |
| Mild cognitive impairment | 13.4 | 12.7 | 14.8 | 0.16 |
| Depression | 8.4 | 8.6 | 7.9 | 0.62 |
| Sarcopenia | 15.7 | 16.9 | 13.3 | 0.03 |
| Frailty | 9.9 | 10.1 | 9.3 | 0.60 |
| Quality of life | 65.5 (14.2) | 66.1 (14.6) | 63.8 (12.9) | <0.01 |
| **Covariates** |  |  |  |  |
| Age | 68.4 (9.5) | 68.3 (9.6) | 68.5 (9.5) | 0.75 |
| Number of chronic conditions | 2.1 (1.6) | 1.9 (1.5) | 2.1 (1.6) | <0.01 |
| Paid job | 25.1 | 26.0 | 23.0 | 0.05 |
| Union status (with partner) | 61.0 | 59.4 | 65.0 | <0.01 |
| Health insurance coverage | 70.0 | 72.7 | 64.8 | <0.01 |

* 2 proportions or t-student tests.

Values in cells are means (std. dev.) or percentages.

**Table S.3. Baseline health and sociodemographic characteristics by wealth, MHAS**

| **Variable** | **Total** | **Q1 (poorer)** | **Q5 (richest)** | **p-value*** |
| --- | --- | --- | --- | --- |
|  | **n=4288** | **n=615** | **n=1583** |  |
| **Outcomes** |  |  |  |  |
| BADL | 4.4 | 2.93 | 3.22 | 0.722 |
| IADL | 3.7 | 4.18 | 3.22 | 0.284 |
| Falls (at least one in last two years) | 33.7 | 37.82 | 30.12 | <0.01 |
| **Covariates** |  |  |  |  |
| Age | 57.6 (6.1) | 58.4(6.8) | 57.5(5.9) | <0.01 |
| Number of chronic conditions | 0.7 (0.8) | 0.5(0.7) | 0.7(0.8) | <0.01 |
| Paid job | 52.3 | 58.1 | 53.2 | 0.04 |
| Union status (with partner) | 75.9 | 73.9 | 79.4 | <0.01 |
| Health insurance coverage | 59.0 | 18.6 | 75.3 | <0.01 |

* 2 proportions or t-student tests.

Values in cells are means (std. dev.) or percentages.

Total is the analytical sample size, not Q1+Q5.

**Table S.4. Baseline health and sociodemographic characteristics by dwelling area, MHAS**

| **Variable** | **Total** | **Rural** | **Urban** | **p-value*** |
| --- | --- | --- | --- | --- |
|  | **n=4288** | **n=858** | **n=3430** |  |
| **Outcomes** |  |  |  |  |
| BADL | 4.4 | 3.4 | 4.7 | 0.10 |
| IADL | 3.7 | 3.9 | 3.7 | 0.70 |
| Falls (at least one in last two years) | 33.7 | 34.7 | 33.5 | 0.51 |
| **Covariates** |  |  |  |  |
| Age | 57.6 (6.1) | 58.2 (6.6) | 57.5 (6) | <0.01 |
| Number of chronic conditions | 0.7 (0.8) | 0.6 (0.8) | 0.7 (0.9) | <0.01 |
| Paid job | 52.3 | 55.1 | 51.6 | 0.07 |
| Union status (with partner) | 75.9 | 81.5 | 74.6 | <0.01 |
| Health insurance coverage | 59.0 | 25.4 | 67.4 | <0.01 |

* 2 proportions or t-student tests.

Values in cells are means (std. dev.) or percentages.

**Table S.5. Measures of wealth (Q5 vs Q1) inequalities in health, SAGE-Mexico**

| **Variable** | **SII** | **95% CI** | **p-value** | **RII** | **95% CI** | **p-value** |
| --- | --- | --- | --- | --- | --- | --- |
| Mild cognitive impairment |  |  |  |  |  |  |
| 2009 | -0.02 | -0.08;0.05 | 0.60 | 0.71 | 0.35;1.41 | 0.34 |
| 2014 | -0.03 | -0.07;0.01 | 0.12 | 0.71 | 0.47;1.06 | 0.09 |
| 2017 | -0.04 | -0.08;-0.01 | 0.04 | 0.71 | 0.51;0.98 | 0.04 |
| 2021 | -0.05 | -0.11;0.01 | 0.06 | 0.70 | 0.45;1.10 | 0.12 |
| Depression |  |  |  |  |  |  |
| 2009 | -0.05 | -0.11;0.01 | 0.06 | 0.52 | 0.23;1.20 | 0.13 |
| 2014 | -0.06 | -0.10;-0.03 | <0.01 | 0.45 | 0.28;0.73 | <0.01 |
| 2017 | -0.07 | -0.10;-0.04 | <0.01 | 0.41 | 0.26;0.63 | <0.01 |
| 2021 | -0.08 | -0.12;-0.04 | <0.01 | 0.36 | 0.20;0.67 | <0.01 |
| Sarcopenia |  |  |  |  |  |  |
| 2009 | -0.08 | -0.15;-0.02 | 0.01 | 0.15 | 0.05;0.40 | <0.01 |
| 2014 | -0.10 | -0.14;-0.06 | <0.01 | 0.20 | 0.11;0.37 | <0.01 |
| 2017 | -0.11 | -0.15;-0.07 | <0.01 | 0.24 | 0.14;0.40 | <0.01 |
| 2021 | -0.12 | -0.17;-0.07 | <0.01 | 0.30 | 0.16;0.57 | <0.01 |
| Frailty |  |  |  |  |  |  |
| 2009 | 0.07 | -0.04;0.18 | 0.22 | 1.28 | 0.95;1.73 | 0.11 |
| 2014 | 0.01 | -0.06;0.06 | 0.94 | 1.02 | 0.86;1.22 | 0.80 |
| 2017 | -0.04 | -0.10;0.02 | 0.22 | 0.89 | 0.76;1.05 | 0.17 |
| 2021 | -0.09 | -0.18;-0.01 | 0.04 | 0.75 | 0.59;0.95 | 0.02 |
| Quality of life |  |  |  |  |  |  |
| 2009 | 7.07 | 4.51:9.62 | <0.01 | 1.12 | 1.08;1.16 | <0.01 |
| 2014 | 7.09 | 5.56;8.63 | <0.01 | 1.12 | 1.09;1.14 | <0.01 |
| 2017 | 7.11 | 5.65;8.56 | <0.01 | 1.12 | 1.09;1.14 | <0.01 |
| 2021 | 7.13 | 5.01;9.24 | <0.01 | 1.12 | 1.08;1.15 | <0.01 |

Slope Inequality Index (SII), and Relative Inequality Index (RII).

Models adjusted for covariates shown in Table 1.

**Table S.6. Measures of sex (female vs male) inequalities in health, SAGE-Mexico**

| **Variable** | **SII** | **95% CI** | **p-value** | **RII** | **95% CI** | **p-value** |
| --- | --- | --- | --- | --- | --- | --- |
| Mild cognitive impairment |  |  |  |  |  |  |
| 2009 | 0.01 | -0.04;0.04 | 0.89 | 1.00 | 0.70;1.45 | 0.98 |
| 2014 | -0.01 | -0.03;0.02 | 0.50 | 0.93 | 0.75;1.16 | 0.53 |
| 2017 | -0.02 | -0.04;0.01 | 0.20 | 0.89 | 0.74;1.08 | 0.24 |
| 2021 | -0.03 | -0.06;0.01 | 0.15 | 0.84 | 0.65;1.09 | 0.19 |
| Depression |  |  |  |  |  |  |
| 2009 | 0.05 | 0.01;0.08 | <0.01 | 3.01 | 1.71;5.27 | <0.01 |
| 2014 | 0.04 | 0.01;0.06 | <0.01 | 2.11 | 1.50;2.96 | <0.01 |
| 2017 | 0.03 | 0.01;0.05 | <0.01 | 1.71 | 1.27;2.29 | <0.01 |
| 2021 | 0.02 | -0.01;0.05 | 0.17 | 1.29 | 0.87;1.90 | 0.21 |
| Sarcopenia |  |  |  |  |  |  |
| 2009 | 0.02 | -0.03;0.06 | 0.45 | 0.97 | 0.57;1.63 | <0.01 |
| 2014 | 0.03 | 0.01;0.06 | 0.03 | 1.23 | 0.85;1.78 | <0.01 |
| 2017 | 0.04 | 0.01;0.07 | <0.01 | 1.42 | 1.01;1.99 | <0.01 |
| 2021 | 0.06 | 0.02; 0.09 | <0.01 | 1.71 | 1.15;2.56 | <0.01 |
| Frailty |  |  |  |  |  |  |
| 2009 | 0.07 | -0.01;0.13 | 0.06 | 1.25 | 1.02;1.51 | 0.03 |
| 2014 | 0.12 | 0.07;0.16 | <0.01 | 1.39 | 1.23;1.57 | <0.01 |
| 2017 | 0.15 | 0.10;0.19 | <0.01 | 1.48 | 1.32;1.66 | <0.01 |
| 2021 | 0.19 | 0.13; 0.25 | <0.01 | 1.61 | 1.37;1.89 | <0.01 |
| Quality of life |  |  |  |  |  |  |
| 2009 | -0.97 | -2.60:0.67 | 0.25 | 0.98 | 0.96;1.01 | 0.24 |
| 2014 | -1.01 | -2.12;0.10 | 0.08 | 0.98 | 0.97;1.01 | 0.07 |
| 2017 | -1.03 | -2.10;0.04 | 0.06 | 0.98 | 0.97;1.01 | 0.06 |
| 2021 | -1.06 | -2.46;0.34 | 0.14 | 0.98 | 0.96;1.01 | 0.14 |

Slope Inequality Index (SII), and Relative Inequality Index (RII).

Models adjusted for covariates shown in Table 1.

**Table S.7. Measures of (rural vs urban) inequalities in health, SAGE-Mexico**

| **Variable** | **SII** | **95% CI** | **p-value** | **RII** | **95% CI** | **p-value** |
| --- | --- | --- | --- | --- | --- | --- |
| Mild cognitive impairment |  |  |  |  |  |  |
| 2009 | -0.02 | -0.06;0.02 | 0.41 | 0.84 | 0.57;1.23 | 0.37 |
| 2014 | -0.01 | -0.04;0.01 | 0.27 | 0.88 | 0.70;1.10 | 0.25 |
| 2017 | -0.01 | -0.04;0.01 | 0.32 | 0.90 | 0.74;1.10 | 0.28 |
| 2021 | -0.01 | -0.05;0.03 | 0.57 | 0.93 | 0.71;1.22 | 0.58 |
| Depression |  |  |  |  |  |  |
| 2009 | -0.02 | -0.06;0.01 | 0.22 | 0.68 | 0.42;1.11 | 0.12 |
| 2014 | -0.01 | -0.03;0.01 | 0.34 | 0.79 | 0.58;1.08 | 0.14 |
| 2017 | -0.01 | -0.03;0.02 | 0.68 | 0.87 | 0.66;1.16 | 0.35 |
| 2021 | -0.01 | -0.03;0.03 | 0.81 | 0.99 | 0.68;1.45 | 0.95 |
| Sarcopenia |  |  |  |  |  |  |
| 2009 | -0.05 | -0.09;-0.01 | 0.04 | 0.64 | 0.37;1.11 | 0.12 |
| 2014 | -0.03 | -0.00;0.01 | 0.06 | 0.77 | 0.52;1.12 | 0.17 |
| 2017 | -0.02 | -0.05;0.01 | 0.18 | 0.85 | 0.60;1.21 | 0.37 |
| 2021 | -0.01 | -0.05; 0.03 | 0.63 | 0.98 | 0.65;1.48 | 0.94 |
| Frailty |  |  |  |  |  |  |
| 2009 | -0.06 | -0.13;0.01 | 0.09 | 0.83 | 0.68;1.00 | 0.05 |
| 2014 | -0.07 | -0.12;-0.03 | <0.01 | 0.83 | 0.73;0.94 | <0.01 |
| 2017 | -0.08 | -0.12;-0.03 | <0.01 | 0.83 | 0.74;0.94 | <0.01 |
| 2021 | -0.09 | -0.15;-0.03 | <0.01 | 0.83 | 0.71;0.98 | 0.03 |
| Quality of life |  |  |  |  |  |  |
| 2009 | 0.11 | -1.54;1.76 | 0.90 | 0.99 | 0.97;1.02 | 0.90 |
| 2014 | 0.35 | -0.79;1.48 | 0.55 | 1.00 | 0.99;1.02 | 0.67 |
| 2017 | 0.49 | -0.62;1.60 | 0.39 | 1.01 | 0.99;1.02 | 0.42 |
| 2021 | 0.68 | -0.77;2.13 | 0.36 | 1.01 | 0.99;1.03 | 0.32 |

Slope Inequality Index (SII), and Relative Inequality Index (RII).

Models adjusted for covariates shown in Table 1.

**Table S.8. Measures of wealth (Q5 vs Q1) inequalities in health, MHAS**

| **Variable** | **SII** | **95% CI** | **p-value** | **RII** | **95% CI** | **p-value** |
| --- | --- | --- | --- | --- | --- | --- |
| Basic activities of daily living |  |  |  |  |  |  |
| 2001 | 0.01 | -0.02;0.03 | 0.70 | 0.56 | 0.39;0.80 | <0.01 |
| 2003 | -0.01 | -0.03;0.02 | 0.67 | 0.56 | 0.41;0.78 | <0.01 |
| 2012 | -0.05 | -0.07;-0.03 | <0.01 | 0.57 | 0.48;0.70 | <0.01 |
| 2015 | -0.06 | -0.08;-0.04 | <0.01 | 0.58 | 0.49;0.70 | <0.01 |
| 2018 | -0.08 | -0.10;-0.06 | <0.01 | 0.58 | 0.49;0.71 | <0.01 |
| 2021 | -0.09 | -0.12;-0.07 | <0.01 | 0.59 | 0.48;0.73 | <0.01 |
| Instrumental activities of daily living |  |  |  |  |  |  |
| 2001 | 0.04 | 0.01;0.07 | <0.01 | 0.82 | 0.51;1.32 | 0.41 |
| 2003 | 0.03 | 0.01;0.05 | 0.035 | 0.80 | 0.53;1.23 | 0.31 |
| 2012 | -0.02 | -0.04;-0.01 | 0.022 | 0.73 | 0.56;0.94 | 0.02 |
| 2015 | -0.04 | -0.06;-0.02 | <0.01 | 0.70 | 0.56;0.90 | <0.01 |
| 2018 | -0.06 | -0.08;-0.03 | <0.01 | 0.68 | 0.54;0.87 | <0.01 |
| 2021 | -0.07 | -0.10;-0.05 | <0.01 | 0.66 | 0.50;0.87 | <0.01 |
| Falls (at least one in last two years) |  |  |  |  |  |  |
| 2001 | -0.10 | -0.13;-0.06 | <0.01 | 0.67 | 0.58;0.78 | <0.01 |
| 2003 | -0.10 | -0.13;-0.06 | <0.01 | 0.67 | 0.59;0.78 | <0.01 |
| 2012 | -0.09 | -0.12;-0.07 | <0.01 | 0.69 | 0.63;0.77 | <0.01 |
| 2015 | -0.09 | -0.12;-0.06 | <0.01 | 0.70 | 0.64;0.78 | <0.01 |
| 2018 | -0.09 | -0.12;-0.06 | <0.01 | 0.71 | 0.64;0.80 | <0.01 |
| 2021 | -0.09 | -0.13;-0.06 | <0.01 | 0.72 | 0.63;0.82 | <0.01 |

Slope Inequality Index (SII), and Relative Inequality Index (RII).

Models adjusted for covariates shown in Table 1.

**Table S.9. Measures of sex (female vs male) inequalities in health, MHAS**

| **Variable** | **SII** | **95% CI** | **p-value** | **RII** | **95% CI** | **p-value** |
| --- | --- | --- | --- | --- | --- | --- |
| Basic activities of daily living |  |  |  |  |  |  |
| 2001 | -0.02 | -0.04;-0.01 | <0.01 | 1.13 | 0.88;1.45 | 0.32 |
| 2003 | -0.01 | -0.03;0.01 | 0.08 | 1.16 | 0.93;1.45 | 0.19 |
| 2012 | 0.03 | 0.02;0.04 | <0.01 | 1.29 | 1.13;1.48 | <0.01 |
| 2015 | 0.04 | 0.03;0.06 | <0.01 | 1.34 | 1.19;1.51 | <0.01 |
| 2018 | 0.05 | 0.04;0.07 | <0.01 | 1.39 | 1.23;1.57 | <0.01 |
| 2021 | 0.07 | 0.06;0.09 | <0.01 | 1.44 | 1.26;1.65 | <0.01 |
| Instrumental activities of daily living |  |  |  |  |  |  |
| 2001 | -0.02 | -0.04;-0.01 | 0.02 | 1.48 | 1.04;2.14 | 0.03 |
| 2003 | -0.01 | -0.03;0.01 | 0.28 | 1.53 | 1.10;2.12 | 0.011 |
| 2012 | 0.04 | 0.03;0.06 | <0.01 | 1.72 | 1.43;2.08 | <0.01 |
| 2015 | 0.06 | 0.05;0.08 | <0.01 | 1.79 | 1.53;2.12 | <0.01 |
| 2018 | 0.08 | 0.07;0.10 | <0.01 | 1.87 | 1.59;2.20 | <0.01 |
| 2021 | 0.10 | 0.09;0.12 | <0.01 | 1.95 | 1.63;2.34 | <0.01 |
| Falls (at least one in last two years) |  |  |  |  |  |  |
| 2001 | 0.16 | 0.14;0.19 | <0.01 | 2.02 | 1.83;2.24 | <0.01 |
| 2003 | 0.16 | 0.14;0.18 | <0.01 | 1.95 | 1.78;2.14 | <0.01 |
| 2012 | 0.13 | 0.12;0.15 | <0.01 | 1.65 | 1.55;1.77 | <0.01 |
| 2015 | 0.12 | 0.11;0.14 | <0.01 | 1.57 | 1.47;1.68 | <0.01 |
| 2018 | 0.11 | 0.10;0.14 | <0.01 | 1.48 | 1.38;1.60 | <0.01 |
| 2021 | 0.10 | 0.09;0.13 | <0.01 | 1.40 | 1.30;1.53 | <0.01 |

Slope Inequality Index (SII), and Relative Inequality Index (RII).

Models adjusted for covariates shown in Table 1.

**Table S.10. Measures of (rural vs. urban) inequalities in health, MHAS**

| **Variable** | **SII** | **95% CI** | **p-value** | **RII** | **95% CI** | **p-value** |
| --- | --- | --- | --- | --- | --- | --- |
| Basic activities of daily living |  |  |  |  |  |  |
| 2001 | -0.01 | -0.04;0.01 | 0.19 | 1.03 | 0.78;1.36 | 0.82 |
| 2003 | -0.01 | -0.03;0.01 | 0.27 | 1.03 | 0.81;1.33 | 0.77 |
| 2012 | 0.01 | -0.01;0.02 | 0.60 | 1.06 | 0.91;1.25 | 0.40 |
| 2015 | 0.01 | -0.01;0.02 | 0.25 | 1.07 | 0.93;1.25 | 0.30 |
| 2018 | 0.01 | 0.01;0.03 | 0.10 | 1.08 | 0.94;1.26 | 0.24 |
| 2021 | 0.01 | 0.01;0.04 | 0.05 | 1.10 | 0.94;1.29 | 0.24 |
| Instrumental activities of daily living |  |  |  |  |  |  |
| 2001 | -0.01 | -0.03;0.01 | 0.21 | 1.08 | 0.79;1.48 | 0.62 |
| 2003 | -0.01 | -0.03;0.01 | 0.30 | 1.08 | 0.81;1.43 | 0.59 |
| 2012 | 0.01 | -0.01;0.02 | 0.56 | 1.07 | 0.91;1.28 | 0.39 |
| 2015 | 0.01 | -0.01;0.02 | 0.24 | 1.07 | 0.92;1.26 | 0.34 |
| 2018 | 0.01 | 0.01;0.03 | 0.10 | 1.07 | 0.92;1.26 | 0.35 |
| 2021 | 0.02 | 0.01;0.04 | 0.05 | 1.07 | 0.90;1.29 | 0.40 |
| Falls (at least one in last two years) |  |  |  |  |  |  |
| 2001 | -0.02 | -0.05;0.01 | 0.16 | 0.92 | 0.82;1.04 | 0.20 |
| 2003 | -0.02 | -0.05;0.01 | 0.17 | 0.93 | 0.84;1.04 | 0.22 |
| 2012 | -0.01 | -0.03;0.01 | 0.46 | 0.97 | 0.9;1.06 | 0.54 |
| 2015 | -0.01 | -0.03;0.02 | 0.69 | 0.98 | 0.91;1.07 | 0.78 |
| 2018 | -0.01 | -0.03;0.02 | 0.95 | 1.00 | 0.92;1.10 | 0.95 |
| 2021 | 0.01 | -0.02;0.03 | 0.83 | 1.01 | 0.92;1.12 | 0.73 |

Slope Inequality Index (SII), and Relative Inequality Index (RII).

Models adjusted for covariates shown in Table 1.

**Table S.11. Measures of inequalities in health by wealth, sex and rural/urban: all-cause mortality rate, MHAS**

| **Variable** | **SII** | **95% CI** | **p-value** | **RII** | **95% CI** | **p-value** |
| --- | --- | --- | --- | --- | --- | --- |
| Wealth (Q5 vs Q1) |  |  |  |  |  |  |
| 2001 |  |  |  |  |  |  |
| 2003 | 0.02 | 0.01;0.02 | 0.68 | 0.88 | 0.78;0.99 | 0.03 |
| 2012 | 0.01 | -0.01;0.01 | 0.29 | 0.93 | 0.86;1.00 | 0.05 |
| 2015 | -0.01 | -0.01;0.01 | 0.84 | 0.94 | 0.87;1.02 | 0.13 |
| 2018 | -0.01 | -0.01; 0.01 | 0.25 | 0.96 | 0.88;1.04 | 0.31 |
| 2021 | -0.01 | -0.02; 0.01 | 0.07 | 0.97 | 0.89;1.07 | 0.59 |
| Sex (female vs male) |  |  |  |  |  |  |
| 2001 |  |  |  |  |  |  |
| 2003 | -0.01 | -0.01;-0.01 | <0.01 | 0.77 | 0.71;0.83 | <0.01 |
| 2012 | -0.02 | -0.02;-0.01 | <0.01 | 0.74 | 0.70;0.78 | <0.01 |
| 2015 | -0.02 | -0.02;-0.01 | <0.01 | 0.73 | 0.70;0.76 | <0.01 |
| 2018 | -0.02 | -0.03;-0.02 | <0.01 | 0.72 | 0.68;0.76 | <0.01 |
| 2021 | -0.02 | -0.03;-0.02 | <0.01 | 0.71 | 0.67;0.75 | <0.01 |
| Area (rural vs urban) |  |  |  |  |  |  |
| 2001 |  |  |  |  |  |  |
| 2003 | -0.01 | -0.02;-0.01 | <0.01 | 0.83 | 0.74;0.92 | <0.01 |
| 2012 | -0.01 | -0.01;-0.01 | <0.01 | 0.88 | 0.82;0.94 | <0.01 |
| 2015 | -0.01 | -0.01;0.01 | <0.01 | 0.89 | 0.84;0.95 | <0.01 |
| 2018 | -0.01 | -0.01; 0.01 | 0.02 | 0.91 | 0.85;0.97 | <0.01 |
| 2021 | -0.01 | -0.01; 0.01 | 0.12 | 0.93 | 0.86;1.00 | 0.05 |

Slope Inequality Index (SII), and Relative Inequality Index (RII).

Models adjusted for covariates shown in Table 1.

No deaths were observed in 2001.

**Table S.12. Sample for all-cause mortality analysis, MHAS study**

|  | **Failure (death year)** | | | | | **Censored** |  |
| --- | --- | --- | --- | --- | --- | --- | --- |
| **Entry** | **2003** | **2012** | **2015** | **2018** | **2021** |  | **Total** |
| 2001 | 526 | 2673 | 1045 | 931 | 1267 | 7151 | 13593 |
| 2003 | 0 | 13 | 4 | 5 | 9 | 49 | 80 |
| 2012 | 0 | 0 | 112 | 149 | 262 | 4649 | 5172 |
| 2015 | 0 | 0 | 0 | 11 | 28 | 421 | 460 |
| 2018 | 0 | 0 | 0 | 0 | 128 | 4032 | 4160 |
| 2021 | 0 | 0 | 0 | 0 | 9 | 347 | 356 |
| Total | 526 | 2686 | 1161 | 1096 | 1703 | 16649 | 23821 |

**Fig. S.1. Flow diagram for the analytical sample, SAGE-Mexico**




Excluded from follow-up (Wave 2)

273 dead

98 losses



Wave 2-2014

n=2651

Follow-up sample=2033

New sample=618



Excluded from follow-up (Wave 3)

242 dead

363 losses



Wave 3-2017

n=2046

Follow-up sample=1791

New sample=255



Excluded from follow-up (Wave 4)

184 dead

255 losses



Wave 4-2021

N=1961

Follow-up sample=1607

New sample=354



Excluded from follow-up sample missing values on some covariates: n=123



Analytical sample (all measurements)

n=1484

**Figure S2. Flow diagram for the analytical sample, MHAS**

Baseline sample (Wave 1-2001)

n=13463

Excluded from follow-up (Wave 2)

526 dead

870 losses

Wave 2-2003

n=12067

Follow-up sample=12067

Excluded from follow-up (Wave 3)

2689 dead

1336 losses

Wave 3-2012

n=13060

Follow-up sample=8042

New sample=5018

Excluded from follow-up (Wave 4)

1160 dead

871 losses

Wave 4-2015

n=11941

Follow-up sample=11029

New sample=912

Excluded from follow-up (Wave 5)

1091 dead

1434 losses

Wave 5-2018

n=13671

Follow-up sample=9416

New sample=4255

Excluded from follow-up (Wave 6)

1688 dead

820 losses

Wave 6-2021

n=12161

Follow-up sample=11163

New sample=998
